# KAT5 histone acetyltransferase mutations in cancer cells

**DOI:** 10.17912/micropub.biology.000676

**Published:** 2022-11-28

**Authors:** Kimberly L Hardison, Tila M Hawk, Renee A Bouley, Ruben C Petreaca

**Affiliations:** 1 The Ohio State University

## Abstract

Cancer cells are characterized by accumulation of mutations due to improperly repaired DNA damage. The DNA double strand break is one of the most severe form of damage and several redundant mechanisms have evolved to facilitate accurate repair. During DNA replication and in mitosis, breaks are primarily repaired by homologous recombination which is facilitated by several genes. Key to this process is the breast cancer susceptibility genes BRCA1 and BRCA2 as well as the accessory RAD52 gene. Proper chromatin remodeling is also essential for repair and the KAT5 histone acetyltransferase facilitates histone removal at the break. Here we undertook a pan cancer analysis to investigate mutations within the KAT5 gene in cancer cells. We employed two standard artificial algorithms to classify mutations as either driver (CHASMPlus algorithm) or pathogenic (VEST4 algorithm). We find that most predicted driver and disease-causing mutations occur in the catalytic site or within key regulatory domains. *In silico* analysis of protein structure using AlphaFold shows that these mutations are likely to destabilize the function of KAT5 or interactions with DNA or its other partners. The data presented here, although preliminary, could be used to inform clinical strategies.

**Figure 1. KAT5 point mutation distribution in cancers:**
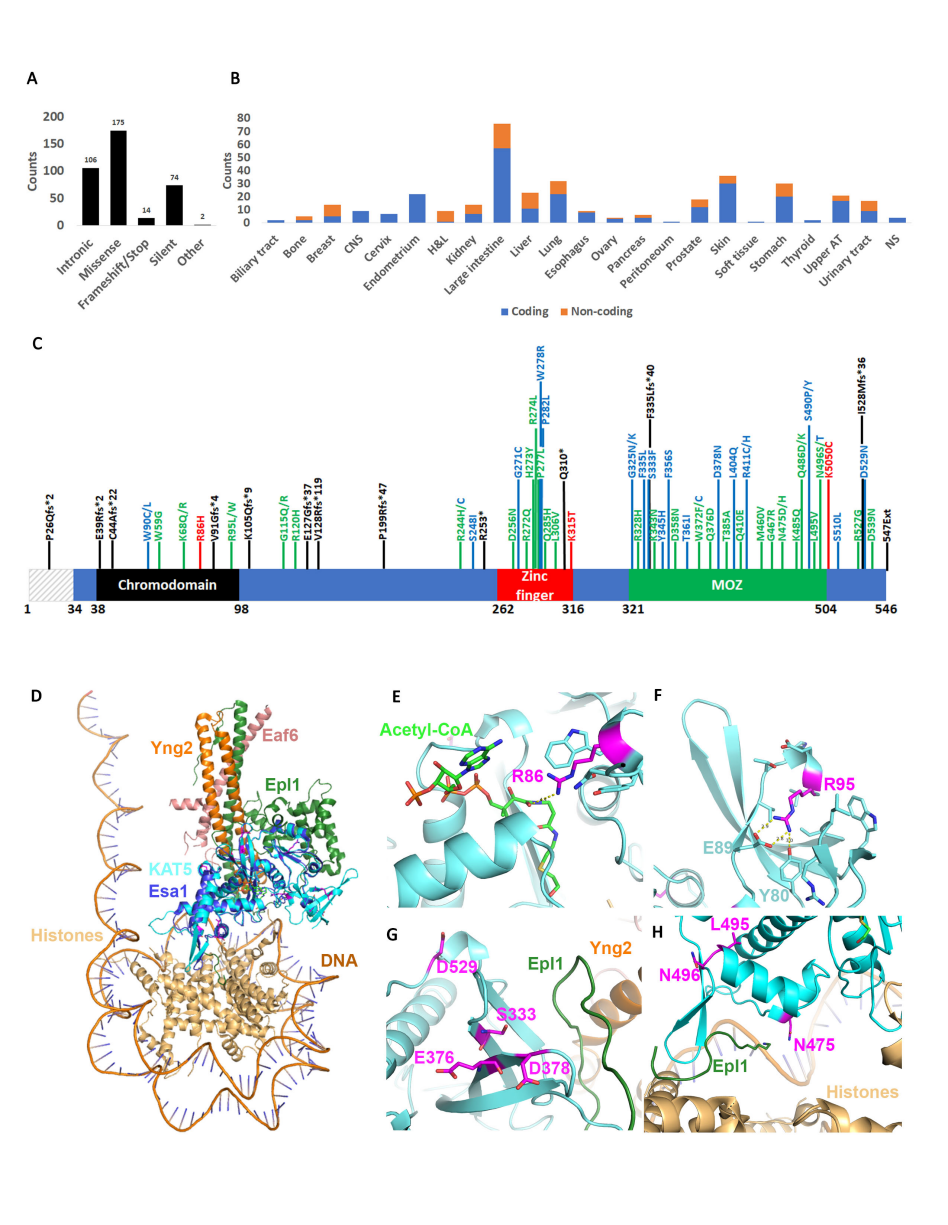
**A** . KAT5 point mutation type in all cancer tissues. **B** . Distribution of coding and non-coding (intronic, 5’, and 3’ UTR) KAT5 mutations by cancer type. **C** . KAT5 mutations with significant (p<0.05) CHASM and VEST p-values. CHASM predicted driver mutations are shown in green, VEST predicted pathogenic mutations are shown in red and mutations that are predicted to be both driver and pathogenic are shown in blue. KAT5 truncating mutations are shown in black. **D.** A model of KAT5 (shown in cyan) was generated in AlphaFold and aligned to the Esa1 chain (shown in dark blue) in a cryo-EM structure of the *S. cerevisiae* NuA4 core complex bound to the nucleosome (PDB ID: 7VVU). The other NuA4 core complex chains are colored as follows: Epl1 in green, Yng2 in orange, and Eaf6 in pink. The histones (H2A, H2B, H3, and H4) are colored in gold. Mutated KAT5 residues are shown in magenta. **E** . The side chain of R86 (R53 of isoform 2) is shown in magenta sticks and is located near the active site and 4.3 Å from acetyl-CoA (shown in bright green) that is bound to the 7VVU structure. **F** . R95 (R71 of isoform 2) is predicted to make interactions with E89 and Y80 to stabilize the tertiary structure of the chromodomain. **G** . Residues S333, E376, D378, and D529 are all clustered in the same region of the protein, which in Esa1 interacts with Epl1 in the NuA4 core complex. **H** . Residues N475, N496, and L495 cluster in a region of the protein that interacts with Epl1 and also facilitates interactions with the histones in the nucleosome.

## Description

Accurate DNA damage repair is essential for genome maintenance and stability as inappropriate repair leads to accumulation of mutations and copy number variations (CNVs). Among the forms of genomic insults, the DNA double strand break (DSB) is one of the most lethal forms of damage because it severs the chromosome into two parts (Audrey et al., 2022). Left unrepaired, it often kills the cell while improper repair can produce small insertions and deletions (InDels) as well as large chromosomal re-arrangements such as inversions, deletions, duplications, translocations, fusions, and other complex architectural changes (Yoshioka et al., 2021). Such large-scale genomic changes are common in cancer cells and can affect global gene expression (Albertson et al., 2003; Cortes-Ciriano et al., 2020; Dahiya et al., 2022; Wang et al., 2020; Yi and Ju, 2018).

DSBs are repaired by two major mechanisms: Non-Homologous End Joining (NHEJ) and Homologous Recombination (HR) (Audrey et al., 2022; Dahiya et al., 2022; Mehta and Haber, 2014). In human cells, NHEJ operates throughout the cell cycle while HR is generally restricted to S-phase and G2/M. The HR restriction to these cell stages is primarily due to the availability of the sister chromatid and the fact that certain HR genes are under the control of the cyclin dependent kinases, primarily CDK1 and CDK2 (Audrey et al., 2022; Kciuk et al., 2022). NHEJ is an error prone repair mechanism as it often results in microdeletions at the site of the lesion (Ghosh and Raghavan, 2021; Paull and Gellert, 1998; Seol et al., 2018). Conversely, HR has been traditionally billed as an error-proof mechanism (Amunugama and Fishel, 2012; Elbakry and Lobrich, 2021; Scully et al., 2019); however, we now know that certain HR pathways are error-prone (Guirouilh-Barbat et al., 2014).

In human cells, a battery of genes including the breast cancer susceptibility genes BRCA1 and BRCA2 work to favor most DSB repair towards error-free pathways (Gorodetska et al., 2019; Li et al., 2019; Roy et al., 2011; Yoshida and Miki, 2004). Central to this form of repair is the loading of the RAD51 recombinase by BRCA2 (assisted by BRCA1 and PALB2) onto resected single stranded DNA to initiate invasion of the sister donor template by the broken chromosome end (Wang et al., 2022). Mutations in the BRCA1/2, RAD51 or PALB2 genes cause improper break repair and pose an increased risk of cancer (Yoshimura et al., 2022). Remarkably, in the absence of BRCA1/2 function, repair relies on RAD52, the central recombinase in yeast but an accessory gene in human cells (Jalan et al., 2019; Lodovichi et al., 2022; Patel et al., 2021; Rossi et al., 2021; Thorpe et al., 2011). RAD52 is able to substitute for the functions of BRCA1/2-PALB2 and load RAD51 to promote strand invasion but this pathway is error prone and leads to chromosomal re-arrangements.

Proper chromatin remodeling is also essential for preventing chromosomal rearrangements (Bakkenist and Kastan, 2015) and numerous factors have been identified that facilitate chromatin remodeling at DSBs (Caron et al., 2021; Karl et al., 2021). KAT5/TIP60 is a histone acetyltransferase belonging to a family of proteins with members in virtually all species studied (Yang, 2004). It has broad functions in DSB repair including acetylation of histone H4 which promotes chromatin remodeling and acetylation of ATM and p53 which results in activation of the DNA damage checkpoint and exchange of phosphorylated histone H2AX with unmodified H2AX (Avvakumov and Cote, 2007a, b; Hong et al., 2015; Lakhter et al., 2013; Wang and Chen, 2010). Changes in KAT5 gene expression has been correlated with cancer progression (Ahmad et al., 2021; Feng et al., 2014; Kim and Lee, 2019; McGuire et al., 2019; Tan et al., 2020; Wei et al., 2019; Yoshida et al., 2011) and small molecule inhibitors targeting KAT5 have already been developed (Brown et al., 2016; Coffey et al., 2012; Cregan et al., 2016; Ghizzoni et al., 2012; Wang et al., 2019). Thus, KAT5 is an important tumor suppressor.

We investigated the distribution of KAT5 point mutations in the reported cancers using the Catalogue of Somatic Mutations in Cancers (COSMIC) (Forbes et al., 2017; Forbes et al., 2015). Most coding mutations were missense or silent while only 14 were frameshift or stop codon (all frameshift mutations introduced a stop codon) ( **Fig.1A** ). Non-coding mutations (intronic, 3’ and 5’ UTR) were also identified. The large intestine was characterized by the highest frequency of mutations reported on COSMIC, but mutations were found in every cancer type, albeit at lower frequency ( **Fig.1B** ).

KAT5 is characterized by a chromodomain at its N-terminus, a zinc finger domain in the middle of the protein and a catalytic domain at its C-terminus ( **Fig.1C** ) (Li and Rasmussen, 2020; Sun et al., 2010). Chromodomains are chromatin interacting modules (Brehm et al., 2004; Taverna et al., 2007) and in the case of KAT5 it interacts with H3K9me3 (Li and Rasmussen, 2020; Sun et al., 2009). Certain mutations in the chromodomain affect KAT5 interaction with chromatin and its enzymatic activity (Sun et al., 2009). The MYST domain includes the catalytic site which can be further differentiated into a zinc finger domain and a HAT domain. MOZ represents a shared domain among a family of histone acetyltransferases named for the *MO* nocytic Leukemia *Z* ink Finger (Perez-Campo et al., 2013). Several post-translational modifications that modulate the function of KAT5 have been identified (Li and Rasmussen, 2020).

At least four different isoforms of KAT5 have been identified (https://www.ncbi.nlm.nih.gov/gene/10524) (Kamine et al., 1996; Li and Rasmussen, 2020; Ran and Pereira-Smith, 2000). Isoform 2 is 513 amino acids and the major KAT5 isoform ( **Fig.1C** ). KAT5 functions as a subunit of a larger complex known as the NuA4 ( *Nu* cleosome *A* cetyltransferase of Histone H *4* ) with roles in both transcription and DSB repair (Allard et al., 1999; Doyon and Cote, 2004; Doyon et al., 2004). In addition to its repair functions, the NuA4 complex also regulates transcription of certain genes. Therefore, KAT5 mutations in cancer cells can affect both DNA damage repair and gene expression.

*Driver and pathogenic KAT5 mutations* . An initial survey of the COSMIC data shows that KAT5 mutations distribute throughout the entire KAT5 coding sequence and although some amino acids are mutated at higher frequency, there is no obvious hotspot. However, not all mutations affect gene function equally. To understand the effect of the reported mutations on gene function, we employed two algorithms: the CHASMplus machine learning algorithm that classifies missense mutations as driver or passenger (Tokheim and Karchin, 2019), and the VEST4 machine learning algorithm that predicts the probability that a mutation is pathogenic (Douville et al., 2016). We used the Open CRAVAT website for this analysis (https://run.opencravat.org) (Masica et al., 2017). Both algorithms generate a p-value for each mutation which can be used to interpret significance. When we categorized those mutations with a significant p-value we found that they cluster primarily in the chromodomain or the active site of the protein ( **Fig.1C** ). This suggests that mutations in the linker region between the chromodomain and the zinc finger or between the zinc finger and the MOZ domain do not have a great effect on gene function. Remarkably, the algorithms predict that a lot of mutations are driver suggesting that altering the function of KAT5 is likely to promote cellular transformation and immortalization. Most driver mutations are also pathogenic. Only three mutations were identified that were pathogenic but not driver ( **Fig.1C** ). Several truncating mutations were also reported ( **Fig.1C** ). The algorithms do not predict the significance of these mutations, but truncating mutations are likely to have a severe effect on gene function.

Of note, we identified a D529N KAT5 mutation that is both driver (p=0.0328) and pathogenic (p=0.0493) that co-occurs with a RAD52 truncation (E320*) in a large intestine metastatic adenocarcinoma. Truncated C-terminus RAD52 alleles have been previously shown to suppress certain germline BRCA2 mutations (Adamson et al., 2020) and the E320* RAD52 truncation also co-occurs with BRCA2 mutations in human cancers (Hamid et al., 2022). Indeed, a BRCA2 E2599* mutation was also present in this patient as well as several BRCA1 mutations. Chromosomal re-arrangements including chromothripsis drive metastasis in colorectal cancers (Kloosterman et al., 2011) and this finding suggests that several repair pathways become destabilized.

*Impact of mutations on protein structure/function .***** To model the impact that KAT5 mutations could have on protein structure and function, an AlphaFold (Jumper et al., 2021) model of human KAT5 was aligned to the recently deposited cryo-EM structure of the *S. cerevisiae* NuA4 core complex bound to the nucleosome (PDB ID: 7VVU) ( **Fig. 1D** ). This showed that the AlphaFold model of human KAT5 aligned very well with the experimentally determined structure of *S. cerevisiae* Esa1 homologue, with an RMSD value of 0.45 Å. AlphaFold was also able to model the N-terminal chromodomain with high confidence, which was not resolved in the cryo-EM structure or a previous crystal structure of the NuA4 core complex (PDB ID: 5J9U) (Xu et al., 2016). This alignment allowed us to predict whether mutations could impact protein-protein interactions within the NuA4 core complex, interactions with the nucleosome, or binding of acetyl-CoA. We identified nine driver or pathogenic mutations that are conserved in *S. cerevisiae* suggesting their evolutionary importance. Based on the protein modeling and alignments, several mutations are predicted to affect KAT5 protein structure and/or function. R86 was modeled with >70% confidence by AlphaFold and is predicted to interact with acetyl-CoA based on the alignments ( **Fig. 1E** ). The R86H mutation would disrupt this interaction. R86 is conserved in yeast, but this residue is not resolved in the published structures, so it is difficult to determine if this predicted interaction is correct. R95 is predicted to mediate tertiary structure interactions in the chromodomain ( **Fig. 1F** ) and a mutation to a hydrophobic and bulky tryptophan residue could disturb the structure of this domain. S333, E376, D378, and D529 all cluster in the same area of the protein ( **Fig. 1G** ). However, only the S333F mutation is hypothesized to influence protein structure due to the hydrophobic nature of phenylalanine. This domain also makes contact with Epl1 in the NuA4 core complex, and thus mutations could affect this interaction. Another cluster of mutations occurs with N475, L495, and N496 ( **Fig.1H** ). The N475 residue specifically is predicted to interact with a lysine residue in a partially resolved Epl1 loop in the cryo-EM structure. This Epl1 loop also mediates interactions with the nucleosome, making this domain particularly important.

*Conclusion* . Numerous KAT5 mutations have been identified in cancer cells and in this work we used established artificial intelligence algorithms to classify them as predicted pathogenic or driver. We further predicted the impact of these mutations through *in silico* modeling. These analyses may inform cancer therapies as driver mutations may be more likely to cause cellular transformation and immortality.

## Methods

Excel files (.csv) with KAT5 mutations were downloaded from COSMIC (https://cancer.sanger.ac.uk/cosmic Version 95 on Monday, April 4 ^th^ , 2022). To extract CHASM and VEST p-values the COSMIC files were processed using OPEN CRAVAT web version (https://www.opencravat.org/).

AlphaFold was used to generate a model of KAT5 isoform 2, which was then aligned to Esa1 in the cryo-EM structure of the *S. cerevisiae* NuA4 core complex bound to the nucleosome (PDB ID: 7VVU) using PyMOL (version 2.3.4). Homologous Esa1 residues were identified using the PyMOL alignment and the protein BLAST sequence alignment.

All figures were made in Photoshop.
